# Effect of optical flow versus attentional strategy on gait in Parkinson's Disease: a study with a portable optical stimulating device

**DOI:** 10.1186/1743-0003-5-3

**Published:** 2008-01-18

**Authors:** Maurizio Ferrarin, Marco Rabuffetti, Mauro Tettamanti, Riccardo Pignatti, Alessandro Mauro, Giovanni Albani

**Affiliations:** 1 Polo Tecnologico, IRCCS S. Maria Nascente, Fondazione Don Carlo Gnocchi Onlus, via Capecelatro, 66 – 20148 Milano, Italy; 2 Laboratorio di Neuropsichiatria geriatrica, Istituto di Ricerche Farmacologiche Mario Negri, Milano, Italy; 3 Divisione di Neurologia e Neuroriabilitazione, Istituto Auxologico Italiano IRCCS, Piancavallo, Verbania, Italy; 4 Dipartimento di Neuroscienze, Università di Torino, Torino, Italy

## Abstract

**Background:**

Several studies have demonstrated the capability of PD subjects to improve gait if appropriate visual cues are provided. Possible explanations referred to attentional factors and to the presence of optic flow on peripheral vision. The aim of the present study was to evaluate separately these two mechanisms in a group of fifteen subjects with Parkinson's Disease at different stages and in a group of ten age-matched controls.

**Methods:**

A microprocessor-controlled portable device implementing two different optical stimulation modalities has been used: bilateral continuous optic flow and unilateral reciprocal optical stimulus that is synchronized to the swing phase of gait. The latter allowed for the implementation of an attentional strategy.

**Results:**

Results showed that mild PD subjects (H&Y<= 2) are responsive to forward oriented optic flow which produces an increment of gait cadence (+ 7.8%) and velocity (+ 8.1%) (p < 0.05), while PD subjects at more advanced stages (H&Y>2) tend to be more responsive to the attentional strategy, through an increase of stride length (+ 19.8%) and a compensatory decrease of cadence (- 16.2%).

**Conclusion:**

Although stated with caution due to the limited number of considered subjects, a possible descriptive model explaining the above findings is proposed, which correlates the different responsiveness to visual stimulation strategies with the progression of pathology and the consequent changes on the activation levels of the involved motor and associative areas.

## Background

Gait in Parkinson's disease (PD) is characterized by short shuffling steps, reduced walking speed and increased stride variability, which increased as a function of the clinical stages of Hoehn and Yahr [1], while cadence does not seem to be affected [2]. Freezing of gait (FOG) is another phenomenon that is common among PD subjects in advanced stages [3], although it is the first symptom of disease in only 7% of cases [4]. Gait disorders can be more pronounced in complex environments that necessitate integration of multiple sensory stimuli.

Several studies have demonstrated that PD subjects can improve gait if appropriate cues are provided, and that the most effective type of cues appear to be visual [2,5]. Different types of visual cues have been tested to facilitate locomotor activity in PD subjects: lines perpendicular to the walking path [6,7], walking sticks with an attached visual cue [8,9]. and laser cueing devices [5] able to project lighting lines on the floor in front of the subject. All of them proved to produce a facilitating effect on gait through an increase of stride length and gait velocity, and, in some patients with FOG, also by reducing the number and duration of freezing episodes [8].

One possible explanation of this phenomenon is provided by an attentional factor [2]: by means of the visual cues, the subject focalizes his attention to the step length thus transforming the automatic movement of gait into a conscious movement. This would induce a facilitation of walking in PD subjects, due to the bypass of the affected neural pathways, i.e. the basal ganglia. In fact, it has been shown that basal ganglia play a primary role in the setting and execution of internally cued sequences of automatic movements [10], but are less involved in the execution of new and complex movements, as demonstrated by PET studies [11], where consciousness level is higher. The external cue could overcome this motor control deficit by activating the associative cortical areas in order to compensate for the hypoactivity of supplementary motor areas secondary to the strio-pallidal-thalamic dysfunction in PD [12]. The enhancement of the locomotor pattern due to an external triggering of each step or to the use of stripes as target for foot positioning, is less convincing because the effects of visual cueing was found to persist for 2 hours after markers removal [2].

A second hypothesis concerning the mechanism of motor facilitation by visual cues, relies on the effects of the optic flow on the peripheral vision produced by the motion of stripes with respect to the walking subject [7]. It has been proposed [13] that a specific visuomotor cerebello-cortical pathways, particularly responsive to rapidly moving targets, is able to by pass the altered functions of basal ganglia in PD subjects. In accordance with this speculation, Majsak et al [14] have found an increase in self-determined maximal speed of reaching movements in PD subjects, when a spatiotemporal visual stimulus of a moving object was provided to the subject. The identification of areas in human visual cortex that respond selectively to fast moving visual cues [15], further support this hypothesis.

The importance of the movement of the visual cues in gait enhancement is underlined by the studies of Azulay et al. [7,16] who found that the facilitating effects of stripes disappeared in presence of stroboscopic lighting, which completely suppress the dynamic component of vision. Additionally, Prokop et al. [17] have found that optic flow modulates walking velocity in normal subjects on the basis of the integration of visual and leg proprioceptive velocity information. Finally, a greater dependency of gait velocity on optic flow was found in PD subjects than in normal age-matched controls [7], implying that their walking velocity relies more on visual than on proprioceptive information, possibly as a consequence of an adaptive process to compensate for the reduced kinesthetic perception found on PD subjects [18].

Other evidence supporting the role of optic flow came from neuropathological and neurophysiological studies which clearly documented a deficit of dopaminergic retinal cells in parkinsonism and PD subjects [19,20] and, consequently, the presence of an abnormal perception of movement [21]. The augmented optic flow provided by horizontal stripes on the ground may compensate for this specific visual deficit.

On the basis of this hypothesis, portable devices able to produce optic flow on the peripheral vision, have been developed [22,23]

In the present study the results of the application of the OSG (Optical Stimulating Glasses) system [23], a microprocessor-controlled portable device based on a compact head-worn display, on a group of 15 PD subjects at different stages of clinical progression and 10 controls are presented and discussed.

In order to explore the possible effects of optic flows and attentional strategies provided by the OSG portable device, two distinct optical stimuli have been considered: a bilateral continuous optic flow in the peripheral field of view and a fixed optical stimulus on each side, synchronized to the swing phase of the homolateral foot.

## Methods

### Subjects

Fifteen subjects with idiopathic Parkinson's Disease and ten healthy subjects (age: 49–60 yrs, height: 150–175 cm, weight: 62–99 kg) voluntarily participated to the study. All had given written informed consent and the protocol had approval from the local Ethical Committee. The clinical characteristics of the subjects at the time of the study, including the Hoehn & Yahr rating (H&Y) and the UPDRS motor score, are summarized in Table 1. All PD subjects were evaluated during on state.

**Table 1:** Details of subjects' characteristics at the time of the study

**Subject**	**Age [yrs]**	**Sex**	**Height [cm]**	**Weight [kg]**	**H&Y Scale**	**UPDRS III Motor score**	**Duration of PD [yrs]**
P1	61	F	153	96	1.5	14	2
P2	64	M	168	81	1.5	17	6
P3	66	M	165	93	1.5	10	8
P4	46	M	174	78	2	12	1
P5	72	F	155	72	2	14	4
P6	76	F	163	64	2	28	4
P7	65	M	182	105	2	35	2
P8	68	F	160	72	3	24	5
P9	59	M	176	76	3	32	7
P10	80	M	172	67	3	40	12
P11	67	M	175	86	3	30	24
P12	73	F	156	63	4	60	15
P13	63	F	163	60	4	35	15
P14	74	M	165	70	4	45	11
P15	65	M	175	108	4	52	3
**PD subjects mean (SD)**	66.6 (8.2)		167 (9)	79.4 (15.2)	2.7 (1.0)	29.9 (15.1)	7.9 (6.4)
**Controls mean (SD)**	56.6 (3.7)		162 (9)	84.9 (13.1)			

### The OSG device

**Figure 1:**
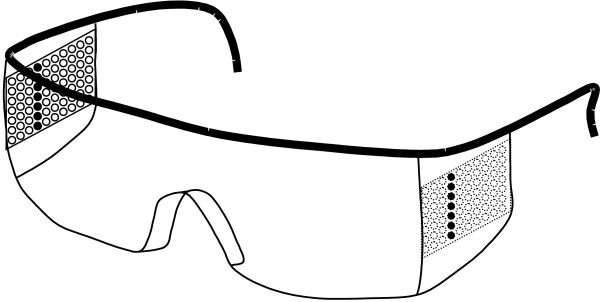
Schematic drawing of the Optical Stimulating Glasses.

The Optical Stimulating Glasses, whose technical specifications are detailed in [23], is a head-worn portable device consisting of a pair of non-corrective protective glasses (Nassau Plus, Aero Ltd), equipped with a matrix display of red light emitting diode (LED) on each side and controlled by a microprocessor (see Fig. 1).

Each display consists of two dot matrixes (dimensions: 12.7 × 17.8 × 6.4 mm; model HDSP703E, Agilent Technologies, Palo Alto, CA, USA) of 5 × 7 LEDs placed side by side. The weight of the head-worn part of the OSG device (glasses, displays and on-board controlling circuits) is 100 gr.

Two foot-switches can be used to synchronize optical stimulation with specific gait event. Different stimulus configuration can be upload to the microprocessor through a host Personal Computer. During the use, the PC is disconnected and the OSG works as a stand-alone device.

The OSG provides a optical stimulation of the peripheral field of view of the subject through two modalities: continuous horizontal optic flow, produced by vertical lighting lines scrolling the matrix displays backward or forward, and lighting stimuli, synchronized to specific step phases. Full technical details of the device have been described previously [23].

In the present study, a foot-switch was positioned under each heel, to provide a signal at the beginning of the stance phase of the corresponding limb, which was known to anticipate the swing phase of the contralateral leg. Therefore, the signal from the right foot-switch was used to activate a stimulus on the left display, so that it would light just before the beginning of the swing phase of the left leg, and vice versa. In this way, an attentional strategy was realized by asking the subject to step as long as possible with one foot, when a light was perceived on the side of that foot. The stimulus ended at contralateral heel off, when the homolateral swinging foot was approaching the ground.

### Experimental procedure

Each subject wore the OSG for a training period of 10–30 min to gain confidence with the device and the different stimulus configurations. Then the subject was requested to stand up from a chair without armrests, to walk straight to a target object placed at a distance of 5 m, to turn around the target object, to return back to the chair and to sit down. The experiments were performed in a large room, with a uniform floor and no external visual or auditory cueing. The subjects were asked to walk comfortably at their natural walking speed.

The trial was repeated in the following randomized four conditions:

a) OSG switched off (Baseline, BL)

b) Bilateral continuous backward optic flow (BOF)

c) Bilateral continuous forward optic flow (FOF)

d) Optical stimulus on each side synchronic to the swing phase of the homolateral leg (attentional strategy, AS).

Optic flows modalities (BOF, FOF) gave the visual effect of a bright vertical line, with a fixed length, scrolling horizontally, forward or backward. Scrolling Speed and Scrolling Delay were set at 40 columns/s and 0.5 s respectively. In the fourth condition (AS), a fixed optical stimulus on the first two columns started on each side just before each step of the homolateral foot. In this case, an attentional task was provided to the subject by requesting him to maximize step length when the stimulus had been perceived on that side.

During the tests, subjects were digitally video recorded (sampling rate = 15 Hz), and from a frame-by-frame analysis of the video, the time of sit-to-stand and the average gait speed, stride length and cadence during straight walking, were computed. The spatio-temporal gait parameters were averaged between back and forth, excluding the turning phase. To ensure data consistency and reliability, all videorecordings were analyzed by the same examiner, who was blinded to test condition.

### Data analysis

Absolute values of stride length and gait velocity have been normalized to the body height (BH) of each subject, to allow inter-subject comparison. Thus, stride length and gait velocity were reported in %BH and %BH/s, respectively.

Descriptive statistics (mean ± SD) were used to summarize results. A nonparametric Wilcoxon Mann Whitney (WMW) test between controls and PD subjects was used to inspect differences in the baseline condition. A nonparametric equivalent of repeated measures analyses of variance (Friedman test), followed by post-hoc Wilcoxon signed rank tests, was used to look for improvement following optical stimulation both on controls and on PD group. The analysis of optical stimulation effect was also performed separately in the two subgroups of subjects with mild (H&Y ≤ 2) and severe (H&Y>2) PD. All tests were two sided. Due to the different strategies (optic flows and attentional strategy) and to the different number of subjects included in the AS condition (data missing in four out of 15 PD subjects for AS), two distinct statistical comparisons have been performed: 1) baseline vs BOF vs FOF, 2) baseline vs AS. The 0.05 level of significance was adopted for main analyses. Post-hoc tests were corrected for multiplicity using Bonferroni criterion. A statistical software for exact nonparametric inference was used (StatXact ver.6, CYTEL Software, Cambridge, MA, USA). Due to the small number of subjects, in addition to statistically significant differences (p < 0.05), also marginally significant (p < 0.10) differences are evidenced in the figures and correspondent p values are reported.

## Results

**Figure 2:**
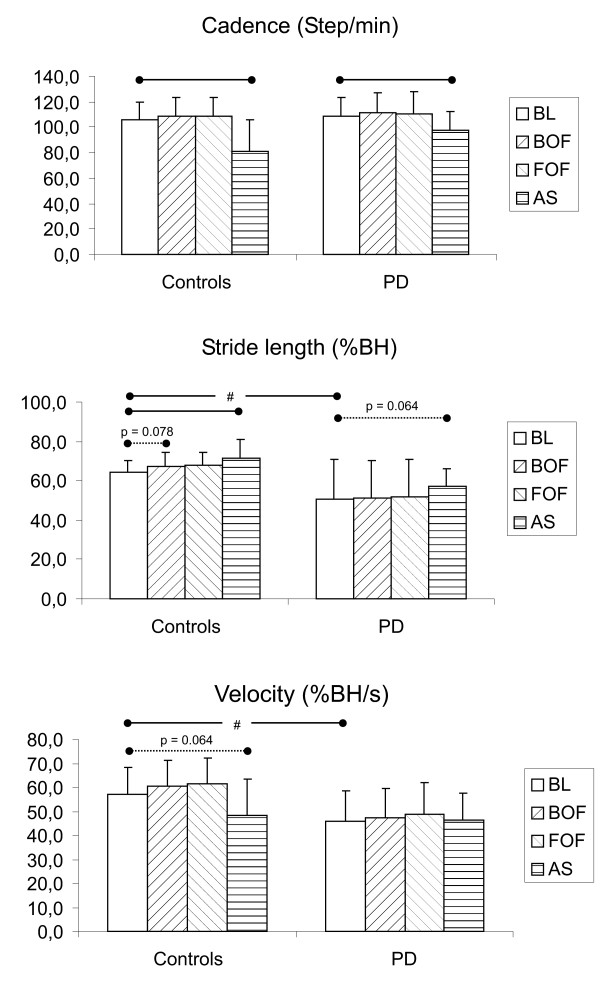
**Gait parameters under different conditions in controls and subjects with Parkinson's Disease**. Comparison of gait velocity, stride length and cadence between the baseline condition (BL), the condition involving optic flow (BOF, FOF) and the condition involving the attentional strategy (AS). The statistical analysis has been performed for each group (controls and PD) separately. Statistically significant differences (p < 0.05) and marginally significant differences (p < 0.10) between conditions are evidenced by, respectively, solid and dotted horizontal lines. # means a significant difference (p < 0.05) of a given parameter between controls and PD group in the baseline condition.

Comparison of gait parameters among the baseline condition (BL), the conditions with continuous optic flow (BOF, FOF) and the condition implying the attentional strategy (AS) are shown for controls and PD subjects in Fig. 2. Table 2 reports mean and standard deviation of numerical values shown in Fig. 2.

**Table 2:** Spatio-temporal gait parameters in the different walking conditions for controls and PD group

**Group**	**Parameter**	**Condition**			
		
		**Baseline**	**BOF**	**FOF**	**AS**
**Control**	**Cadence [step/min]**	105.9 (13.4)	108.2 (15.2)	108.6 (15.3)	81.4 (24.7)
	**Stride length [%BH]**	64.2 (6.3)	67.3 (7.3)	67.7 (6.5)	71.3 (9.7)
	**Velocity [%BH/s]**	57.0 (11.0)	60.7 (10.7)	61.4 (11.0)	48.1 (15.1)
**PD group**	**Cadence [step/min]**	109.1 (14.7)	111.0 (16.1)	111.0 (16.9)	97.2 (15.2)
	**Stride length [%BH]**	50.5 (20.3)	51.2 (19.0)	51.8 (19.2)	57.1 (9.2)
	**Velocity [%BH/s]**	45.8 (12.9)	47.5 (12.1)	48.8 (13.3)	46.4 (11.3)

Mean values and SD (in brackets). Statistically significant differences between conditions and baseline are reported in fig. 2. BOF = backward optic flow, FOF = forward optic flow, AS = attentional strategy. BH = body height.

In the baseline condition, when subjects wore the OSG but the device was switched off, PD subjects walked significantly slower (45.8 ± 12.9 %BH/s) than controls (57.0 ± 11.0 %BH/s) due to a shorter stride length (50.5 ± 20.3 vs 64.2 ± 6.3 %BH), while cadence was only slightly and not significantly increased (109.1 ± 14.7 vs 105.9 ± 13.4 step/min).

With continuous optic flows, control subjects tended to increase (although not significantly) gait velocities respect to the baseline condition, because of a slight increase of stride length, while cadence was not affected. Conversely, with the attentional strategy stride length increased while cadence reduced significantly, resulting in a marginally significant reduction of gait velocity.

PD subjects, as a group, presented trends similar to controls, although without any statistical significance in differences among conditions, except for a reduction of cadence (and a concomitant marginally significant increase of stride length) in the AS condition respect to basal.

**Figure 3:**
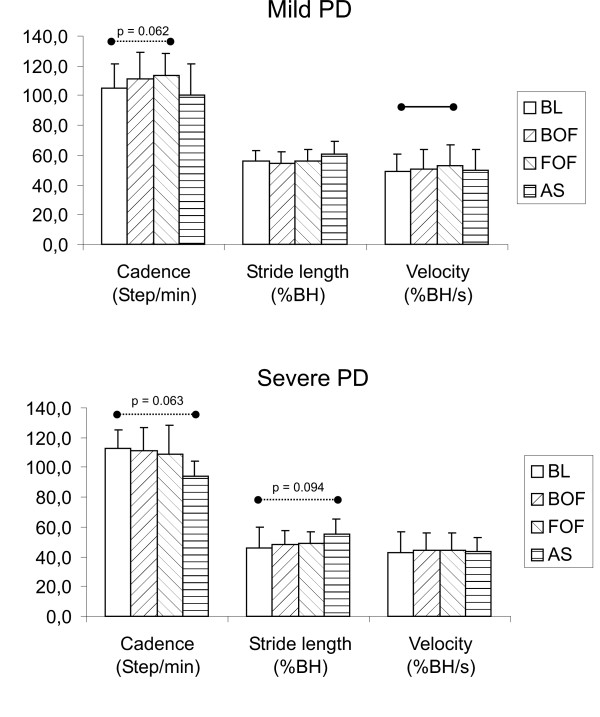
**Gait parameters under different conditions in subjects with mild and severe Parkinson's Disease**. Comparison of gait velocity, stride length and cadence between the baseline condition (BL), the conditions involving optic flow (BOF, FOF) and the condition involving the attentional strategy (AS) for the subgroup of mild PD (H&Y≤2) and severe PD (H&Y>2) subjects. The analysis has been performed for each group separately. Statistically significant differences (p < 0.05) and marginally significant differences (p < 0.10) between conditions are evidenced by, respectively, solid and dotted horizontal lines.

Interestingly, disease severity correlated with stimulation effects: PD subjects at a H&Y stage ≤ 2 disclosed, as a group, different responses to optical stimuli respect those patients at a more advanced stages (H&Y>2). In particular, as shown in Fig. 3, subjects with mild PD did not significantly change stride length in any conditions compared to the baseline, while velocity showed a significant increase with forward-oriented optic flow (due to a marginally significant increase of cadence) but not with the attentional strategy. Conversely, severe PD subjects did not significantly change gait parameters respect to basal walking in any condition involving an optic flow, while the attentional strategy induced a marginally significant increase of stride length and a concomitant reduction in step cadence, with almost no change in gait velocity. Table 2 reports mean and standard deviation of numerical values shown in Fig. 3.

**Figure 4:**
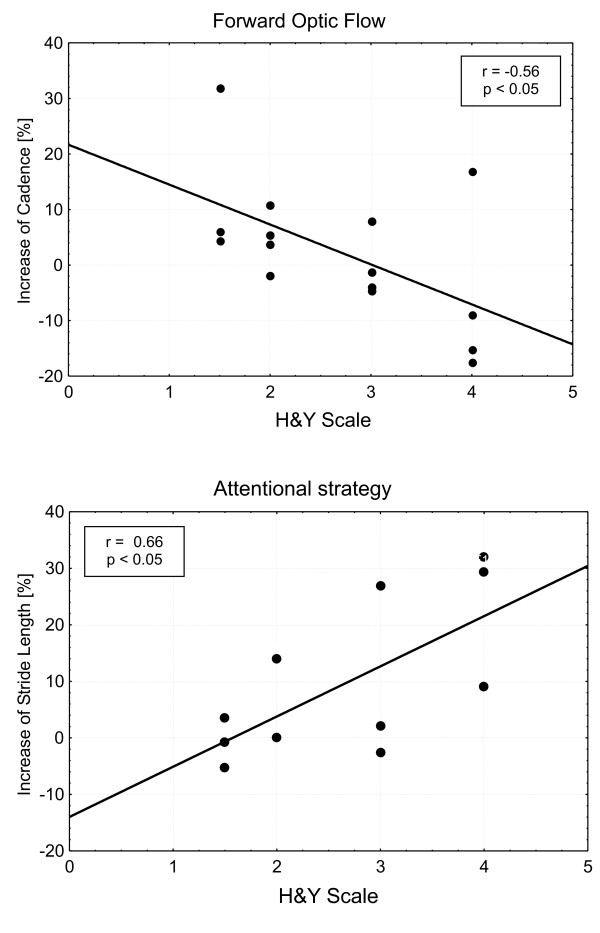
**Effect of visual stimuli vs PD severity**. Correlation between the effect of visual stimuli and PD severity for the whole group of PD subjects. Increase of gait cadence in the forward optic flow condition (above) and increase of stride length with the attentional strategy (below). The changes are computed as the percentage increase of cadence or stride length measured in the considered condition respect that measured in the basal condition. Least square linear regression lines are superimposed. Spearman rank correlation coefficients (r) are reported with significance level.

In Fig. 4 the effects of visual stimuli on the affected gait parameters (cadence for forward optic flow and stride length for attentional strategy) are plotted versus disease severity, showing opposite trends: as disease severity worsens, the effect of optic flow decreases while that of attentional strategy increases. The Spearman rank order correlation coefficients are respectively r = -0.56 (p < 0.05) and r = 0.66 (p < 0.05).

## Discussion

The results of the present study showed that, in the analyzed PD subjects, severity of the disease correlates with the different effects of the two visual stimulation modalities considered: forward-oriented optic flow ameliorates velocity and, slightly, cadence in subjects with mild PD, while the attentional strategy induces a slight increase of stride length and a decrease of cadence, with no changes in gait velocity, in subjects with severe PD.

The different responsiveness of PD subjects to the optic flow might be ascribed, in part, to the slight different walking behavior they already present in the baseline condition: severe PD subjects, which walk at a slower velocity and slightly higher cadence than mild PD subjects (see Table 3), may not be able to further increase cadence, possibly due to a protective mechanism to prevent freezing or festination [24]. Moreover, the optic flow velocity may interfere with PD subjects in a different way because of their different gait velocity: a given scrolling velocity may be optimal for mild PD subjects but too fast for severe subjects, who walk slower. Anyway, those slight differences in the baseline condition may not be enough to explain the obtained results, which have shown to correlate stimuli effect and disease severity.

**Table 3:** Spatio-temporal gait parameters in the different walking conditions for mild and severe subgroups of PD subjects

**Group**	**Parameter**	**Condition**			
		
		**Baseline**	**BOF**	**FOF**	**AS**
**Mild PD**	**Cadence [step/min]**	105.1 (16.6)	110.8 (17.9)	113.3 (15.0)	100.2 (20.8)
	**Stride length [%BH]**	55.8 (7.3)	54.8 (7.7)	55.7 (7.8)	60.4 (8.9)
	**Velocity [%BH/s]**	49.2 (11.8)	50.9 (13.0)	53.2 (13.3)	50.1 (13.4)
**Severe PD**	**Cadence [step/min]**	112.6 (12.8)	111.2 (15.5)	108.9 (19.2)	94.4 (10.1)
	**Stride length [%BH]**	45.9 (14.1)	48.0 (9.3)	48.8 (7.9)	55.0 (10.3)
	**Velocity [%BH/s]**	42.8 (13.8)	44.6 (11.3)	44.5 (11.5)	43.3 (9.3)

Mean values and SD (in brackets). Statistically significant differences between conditions and baseline are reported in fig. 3. BOF = backward optic flow, FOF = forward optic flow, AS = attentional strategy. BH = body height.

An additional explanation might be related to the preserved dopaminergic function of retinal system in PD subjects at earlier stages compared with that of subjects at advanced stages. There is evidence suggesting that the activity of dopaminergic neurons of retina is affected in PD and it is influenced by the severity of disease, motor status and therapy: indeed, there is a correlation between neurophysiological impairment of retina both with severity of disease [25] and l-dopa or dopamine receptors blocker therapy [26]; furthermore, it was also shown that vision fluctuates in parallel with motor fluctuations [27]. In favor of this interpretation, which correlates the degree of integrity of dopaminergic neurons of retina and locomotor response to the optic flow stimulation, there is the result that also normal subjects (thus with normal dopaminergic function), in our study, are influenced by optic flow, showing a nearly significant increase of stride length.

Finally, regarding the pathophysiological model of altered gait in PD, in favor of the hypothesis of the involvement of other systems in addition to basal ganglia, there is increasing clinical evidence of a reduction of the positive effects on gait in the long term treatment with Deep Brain Stimulation of subthalamic nucleus.

The tendency of subjects with severe PD to behave like controls in response to an attentional stimulus (increasing stride length, decreasing cadence), would suggest that, in advanced stages of disease, as a consequence of progression of motor symptoms, subjects are paradoxically more receptive to the attentional triggers than subjects in earlier stages, who are not able to significantly change their gait parameters inside an attentional strategy.

Similar results have been recently reported by Van Wegen et al. [28], who found that attentional stimulation (rhythmic visual cues) seems to ameliorate stride parameters more in PD subjects with high disease severity (patients medicated) than in denovo patients. In this work the role of optic flow (which did not evoked any significant variation on gait) was methodologically different from our study, because it was conceived from the perspective of a potential suppressive action on visual cues effects.

**Figure 5:**
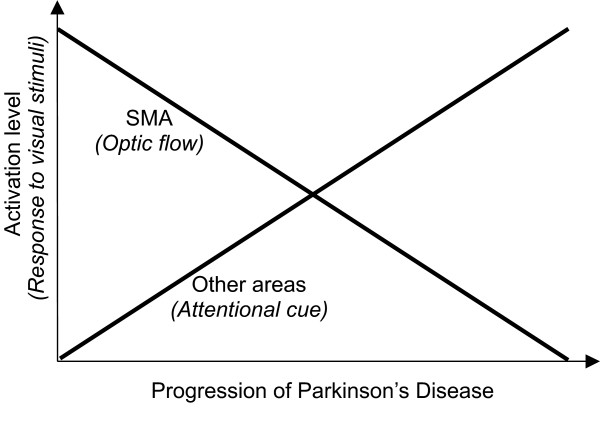
**Schematization of responsiveness of PD subjects to visual stimuli**. The diagram represents schematically the responsiveness of PD subjects to different visual stimuli, as a consequence of activation levels of supplementary motor area and other areas (associative, sensory), versus disease progression.

Considering the global behavior of the PD subjects involved in this study during basal, attentional and optic flow conditions, an integrative model can be hypothesized, as shown in Fig. 5. In this model, the activation level of other cortical areas (associative, sensory), inside the loop compensatory of the decline of supplementary motor area secondary to nigrostriatal dysfunction [12,29], is considered as function of progression of the disease. This, in turn, influences the motor response to attentional cue and to optic flow.

At the present stage, the above model is to be considered only a description of behavioural trend concerning the locomotor response; thus, a rigid correlation between severity of disease and locomotor response to optic flow may not always be expected. Indeed, the progression of degeneration of dopaminergic activity may not be necessarily homogeneous, and in some cases, contrary to the group average, signs of preserved activity of dopaminergic retinal system can be found, in spite of signs of a damaged dopaminergic motor system.

From a rehabilitative point of view, we argue that although encouraging results were found in particular on mild PD subjects with forward optic flow stimulation, wider clinical trials, with additional training sessions must be performed, before a conclusion can be drawn on the efficacy of the OSG device as an orthotic aid for gait and on the feasibility of its use in not-supervised conditions. However, it is foreseen that the most effective optical stimulation strategy should be identified for each subject and that it may change during the progression of the disease, highlighting the need of a programmable and customisable optical stimulating device. In particular, the choice of optimal optic flow velocity, which may be related to subject speed, could be a crucial aspect for system efficacy and should be considered in future works. In this respect, foot switches can be used to pace the optic flow velocity as a function of walking speed. An additional future development of the present study should be the adoption of a more accurate approach for the assessment of gait performances, by means of wearable or laboratory-based motion capture systems, able to highlight subtle but relevant changes in gait behaviours, like stride-to-stride variability and kinematics/kinetics patterns.

## Conclusion

The results of the present study suggest that gait behaviours of PD subjects can be influenced by optical stimulation provided by portable devices, like the Optical Stimulating Glasses, although their effectiveness as walking aids should be confirmed in wider clinical studies. The different effects of continuous optic flow and optically-mediated attentional strategies on walking parameters (cadence and stride length) appear to be correlated with disease's severity: mild PD subjects are more receptive to optic flow, while severe PD subjects to the attentional strategy. A possible interpretation of these findings refers to the deterioration of dopaminergic function of the retinal system as well as to changes in the activation level of involved motor and associative areas, related to pathology progression.

## Competing interests

The author(s) declare that they have no competing interests.

## Authors' contributions

MF conceived and coordinated the study, participated in data collection and analysis and drafted the manuscript. MR and RP participated in the design of the study, data analysis and helped to draft the manuscript. MT participated in data analysis and performed the statistical analysis. AM participated in the design of the study and data analysis. GA participated in the design of the study and in drafting the manuscript, performed subjects selection and conceived the pathophysiological model for data interpretation. All authors read and approved the final manuscript.
